# Systemic Inflammation Across Metabolic Obesity Phenotypes: A Cross-Sectional Study of Korean Adults Using High-Sensitivity C-Reactive Protein as a Biomarker

**DOI:** 10.3390/ijms252111540

**Published:** 2024-10-27

**Authors:** Seong-Uk Baek, Jin-Ha Yoon

**Affiliations:** 1 Graduate School, Yonsei University College of Medicine, Seoul 03722, Republic of Korea; 2 Department of Preventive Medicine, Yonsei University College of Medicine, Seoul 03722, Republic of Korea

**Keywords:** adiposity, body weight, metabolic syndrome, oxidative stress

## Abstract

Chronic systemic inflammation is a hallmark of obesity. This cross-sectional study aimed to investigate the association between metabolic obesity phenotypes and inflammatory markers in Korean adults (*N* = 21,112; mean age: 50.9 ± 16.6). Metabolic obesity phenotypes were categorized into metabolically healthy non-obesity (MHNO), metabolically unhealthy non-obesity (MUNO), metabolically healthy obesity (MHO), and metabolically unhealthy obesity (MUO) based on body mass index and the presence of any metabolic abnormalities. High-sensitivity C-reactive protein (hs-CRP) levels were measured. Multiple linear regression was used to determine the association between obesity phenotypes and hs-CRP levels. In the male sample, compared to the MHNO type, the MUNO, MHO, and MUO types were associated with a 22.3% (95% confidence interval; CI: 14.7–30.3%), 15.8% (95% CI: 2.6–30.7%), and 12.5% (95% CI: 3.0–22.9%) increase in the hs-CRP levels, respectively. The association between metabolic obesity types and hs-CRP levels was stronger among the female sample; compared to the MHNO type, the MUNO, MHO, and MUO types were associated with a 30.2% (95% CI: 22.8–38.2%), 16.0% (95% CI: 6.5–26.4%), and 22.8% (95% CI: 13.6–32.8%) increase in the hs-CRP levels, respectively. Our findings indicate a varying profile of systemic inflammation across different metabolic obesity phenotypes.

## 1. Introduction

Obesity is a pressing global public health concern [1,2], resulting in substantial health and economic burdens. South Korea has also experienced a gradual increase in obesity prevalence over the past decade, with 27.8% of women and 49.2% of men classified as obese by 2021 [3]. Obesity is associated with increased risks of developing cardiovascular disease (CVD) [4,5] and overall mortality [6,7].

Inflammatory response is a hallmark of obesity. Adipose tissue (AT) serves as the primary site of fat storage and is composed of various cell types, including adipocytes, pre-adipocytes, fibroblasts, and immune cells [8]. Obesity can induce low-grade chronic inflammation by altering immune cells within adipose tissue (AT). AT macrophages (ATMs) undergo significant phenotypic changes during obesity, accompanied by an increase in their overall numbers. M1-polarized ATMs release proinflammatory cytokines, such as interleukin-6 (IL-6) and tumor necrosis factor-α (TNF-α) [9]. The inflammatory response triggered by obesity plays a critical role in the occurrence of insulin resistance and type 2 diabetes [10,11]. C-reactive protein (CRP) is a widely used inflammatory marker of hepatic origin whose levels are elevated in response to IL-6 secretion [12]. Obesity is associated with the accumulation of free fatty acids in adipose tissue, which can activate proinflammatory serine kinase cascades, including *IKK*β and *JNK1* [13,14]. These signaling pathways enhance the release of IL-6, which subsequently induces the hepatic synthesis of CRP [15,16]. A meta-analysis demonstrated that body mass index (BMI) is positively correlated with log-transformed CRP levels [17]. In addition, close associations between obesity and CRP levels have been documented in several population-based studies [18,19,20].

Obese individuals are not a homogeneous group; this concept highlights the importance of identifying “metabolically healthy” obese individuals [21,22]. Studies have categorized metabolic obesity phenotypes based on metabolic dysfunction status. Although there is no universally accepted definition, metabolically healthy obesity (MHO) is often defined as having a BMI classified as obese but not meeting the criteria for metabolic syndrome (MetS) or any of its components [23]. While the prevalence of MHO exhibits large regional differences, approximately 10–30% of obesity cases are MHO [24]. MHO is reported to have more favorable health outcomes than metabolically unhealthy obesity (MUO). Studies have demonstrated the distinct characteristics of MHO and MUO, for example, MHO is characterized by less ectopic fat, including hepatic and visceral fat, and more leg fat than MUO [22,25,26]. Additionally, unlike MUO; which exhibits adipocyte hypertrophy, MHO exhibits adipocyte hyperplasia and relatively intact insulin sensitivity [22,25,26].

While the relatively benign characteristics of MHO compared to those of MUO are not yet fully understood, one key factor is the difference in the inflammatory response between MHO and MUO. AT in individuals with the MHO phenotype is characterized by lower macrophage infiltration and lower levels of inflammation [25,27], which may play a significant role in the protective metabolic profile observed in this phenotype. However, the inflammatory response in MHO compared to that in MUO or non-obese individuals remains controversial. Several studies have reported lower CRP levels [28,29,30] in MHO than in MUO; however, other studies have found no significant differences in inflammatory markers between the two groups [31,32,33]. For instance, there were no significant differences in inflammatory markers, such as IL-1β, IL-6, or TNF-α, or inflammatory gene expression between MHO and MUO [33].

The variations in the inflammatory responses based on the sex across metabolic obesity phenotypes also remain underexplored. Prior research has demonstrated that the association between obesity and CRP levels is particularly pronounced in women [17,34]. Although the exact mechanisms remain unclear, the proposed explanations include differences in the metabolic activity of adipose tissue, leptin levels, and a higher percentage of body fat in women [17,34,35]. However, evidence regarding how the association between metabolic obesity phenotypes and inflammatory markers differs according to sex remains scarce. Therefore, this study examined the systemic inflammation across metabolic obesity phenotypes among Korean adults. Our hypotheses were as follows: (i) hs-CRP levels are higher in individuals with MHO and MUO compared to those with metabolically healthy non-obesity (MHNO). (ii) hs-CRP levels are higher in the MUO group than in the MHO group. (iii) A significant sex difference exists in the association between the MUO phenotype and hs-CRP levels, with more pronounced associations observed in women.

## 2. Results

### 2.1. Demographic Features of This Study Participants

Table 1 displays the sociodemographic features of the sample. The sample consisted of 9250 (43.8%) men and 11,862 women (56.2%). The mean (standard deviation) of age was 50.9 (16.6) among the study participants. The median (Q1, Q3) BMI was 23.7 (21.5, 26.0) in the sample.

### 2.2. Distribution of hs-CRP by the Metabolic Obesity Phenotypes

**Figure 1:**
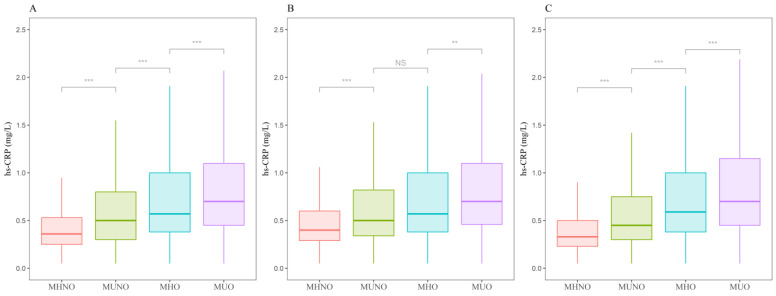
Distribution of hs-CRP (high-sensitivity C-reactive protein) values according to the metabolic obesity phenotypes (MHNO, metabolically healthy non-obesity; MUNO, metabolically unhealthy non-obesity; MHO, metabolically healthy obesity; and MUO, metabolically unhealthy obesity). (**A**) Median (Q1, Q3) values of hs-CRP in the overall sample; (**B**) median (Q1, Q3) values of hs-CRP in the male sample; and (**C**) median (Q1, Q3) values of hs-CRP in the female sample. Wilcoxon rank sum test with Bonferroni correction for multiple comparisons was employed (** *p* < 0.01, *** *p* < 0.001, NS: non-significant differences).

Figure 1 shows the distribution of hs-CRP according to metabolic obesity phenotypes. The median (Q1, Q3) of hs-CRP level values were 0.54 (0.33, 1.05) among the overall sample, and the median (Q1, Q3) of hs-CRP level (mg/L) according to the metabolic obesity phenotypes was 0.38 (0.26, 0.60), 0.51 (0.33, 0.99), 0.61 (0.40, 1.20), and 0.80 (0.50, 1.60) for MHNO, metabolically unhealthy non-obesity (MUNO), MHO, and MUO, respectively (Figure 1A). The median hs-CRP level (mg/L) among the men was 0.40, 0.60, 0.60, and 0.80 for MHNO, MHO, MUNO, and MUO, respectively (Figure 1B). The median hs-CRP (mg/L) among the women was 0.35, 0.50, 0.63, and 0.85 for MHNO, MUNO, MHO, and MUO, respectively (Figure 1C).

**Figure 2:**
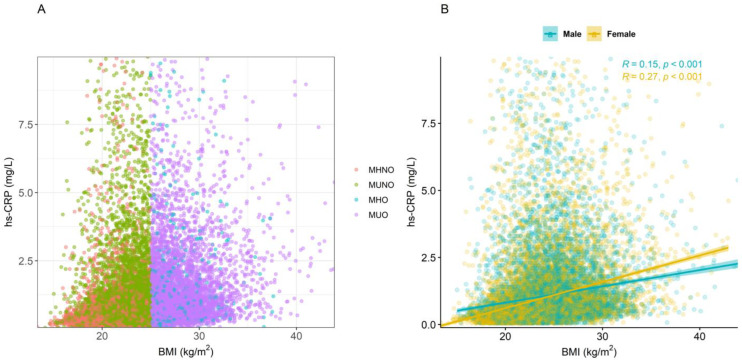
Scatter plot of body mass index (BMI) and hs-CRP (high-sensitivity C-reactive protein) levels. (**A**) Scatter plot of BMI and hs-CRP levels according to metabolic obesity phenotypes (MHNO, metabolically healthy non-obesity; MUNO, metabolically unhealthy non-obesity; MHO, metabolically healthy obesity; and MUO, metabolically unhealthy obesity); (**B**) scatter plot of BMI and hs-CRP levels based on sex. Pearson’s correlation coefficients were presented.

Figure 2A shows distributions of BMI (kg/m^2^) and hs-CRP level (mg/L) according to the metabolic obesity phenotypes (Figure 2). Figure 2B shows the distribution of BMI and hs-CRP levels according to sex. The correlation coefficients between BMI and hs-CRP were 0.15 (*p* < 0.001) in men and 0.27 (*p* < 0.001) in women.

### 2.3. Association Between Metabolic Obesity Phenotypes and hs-CRP

Table 2 displays the results of the log-linear models of the association between the metabolic obesity phenotypes and hs-CRP levels in the overall sample. After adjusting for confounders, compared to the MHNO type, the MUNO, MHO, and MUO types were associated with a 29.3% (95% CI: 24.1–34.6%), 15.4% (95% CI: 6.4–25.2%), and 23.3% (95% CI: 16.4–30.5%) increase in the hs-CRP levels, respectively. When stratified by obesity, MUO is associated with a 14.1% (95% CI: 5.5–23.4%) increase in hs-CRP level compared to those in the MHO type. In the model including the interaction terms between sex and metabolic obesity phenotypes (Table S1), a significant multiplicative interaction between female and MUO type on an increase in the hs-CRP level was observed at *p* < 0.001.

Table 3 shows sex-stratified analyses of the association between the metabolic obesity phenotypes and hs-CRP levels. In the male sample, compared to the MHNO type, the MUNO, MHO, and MUO types were associated with a 22.3% (95% CI: 14.7–30.3%), 15.8% (95% CI: 2.6–30.7%), and 12.5% (95% CI: 3.0–22.9%) increase in the hs-CRP levels, respectively. The association between metabolic obesity types and hs-CRP was stronger among the female sample; compared to the MHNO type, the MUNO, MHO, and MUO types were associated with a 30.2% (95% CI: 22.8–38.2%), 16.0% (95% CI: 6.5–26.4%), and 22.8% (95% CI: 13.6–32.8%) increase in the hs-CRP levels, respectively. When stratified by obesity, compared to MHO, MUO was associated with a respective increase of 0.4% (95% CI: −10.2–12.1%) and 38.7% (24.0–55.2%) in the hs-CRP levels in the men and women.

Table S2 shows the association between metabolic obesity types and high CVD risk (hs-CRP ≥ 3 mg/L) on multivariate logistic regression models. The association between MUO and elevated hs-CRP levels was stronger in women than in men, which is consistent with the main analyses. Table S3 shows the association between the metabolic obesity phenotypes and hs-CRP levels based on alternative classification criteria, which is consistent with the results of the main analyses. Tables S4 and S5 show the results of the analyses without adjustment for BMI in the regression models. Compared to the MHNO type, the MUNO, MHO, and MUO types were associated with a 42.7% (95% CI: 37.0–48.6%), 89.7% (95% CI: 75.7–104.9%), and 114.9% (95% CI: 105.9–124.4%) increase in the hs-CRP levels, respectively (Table S4). The associations between obesity phenotypes and hs-CRP levels were stronger in women than in men (Table S5).

## 3. Discussion

This study aimed to examine systemic inflammation across the four metabolic obesity phenotypes and found that the MUO type was associated with elevated hs-CRP levels in comparison to both MHNO and MHO phenotypes. In addition, significant sex-based differences were observed, with the increase in hs-CRP levels being more pronounced in women than in men in the MUO group. Our findings provide evidence from a large-scale population-based sample on how hs-CRP levels are elevated in MHO and MUO, overcoming the limitations of previous studies with smaller sample sizes that investigated the association between metabolic obesity phenotypes and inflammatory markers.

The hs-CRP, a sensitive biomarker of inflammation, is a routine tool in clinical practice for diagnosing acute inflammatory states; however, the prognostic value of the hs-CRP level in predicting CVD events and outcomes has been robustly supported by meta-analytic evidence, particularly in the context of chronic low-grade systemic inflammation [36,37,38]. These findings suggest that prevention and treatment strategies, including lifestyle modifications, such as weight reduction, are crucial for mitigating CVD risk in individuals with obesity. Although MHO has previously been characterized by a favorable metabolic profile [39], our results indicate that even this group exhibits significantly elevated hs-CRP levels compared to that in the MHNO type. These findings highlight the importance of managing obesity, even in cases of MHO, which is often perceived as “benign obesity.” A previous study demonstrated that the implementation of lifestyle modifications over one year among individuals with MHO significantly reduced the levels of inflammatory markers, including CRP [40].

There have been inconsistent findings regarding the inflammatory markers between the MHO and MUO phenotypes. A few studies have reported no significant differences in inflammatory markers or gene expression between individuals with MHO and MUO [31,33]. Additionally, in a population-based study conducted in Brazil, the levels of inflammatory markers in individuals with MHO were not significantly higher than those in individuals with MHNO [41]. Conversely, our study supports other research, including a meta-analysis by Su et al. (2024), which suggested that the inflammatory markers, such as hs-CRP, are lower in the MHO group than in the MUO group but higher in MHNO individuals [28,42,43]. Notably, our study utilized a large-scale population-based sample, offering a significant advantage over previous studies that relied on smaller sample sizes.

Studies have indicated that MHO and MUO exhibit distinct characteristics, including AT growth, ATM profiles, fat distribution, and genetic predisposition, which may contribute to higher hs-CRP levels in MUO. MHO and MUO are distinguished based on adipocyte hypertrophy and hyperplasia, respectively [44]. In contrast to adipocyte hyperplasia, adipocyte hypertrophy is often accompanied by inefficient remodeling of the extracellular matrix, characterized by inadequate vascularization, resulting in hypoxia and the release of pro-inflammatory cytokines [45]. MHO is associated with a distinct ATM profile compared to MUO, which is marked by a higher M2:M1 ratio [46]. Additionally, the body fat distribution patterns differed between the MHO and MUO groups. Individuals with MUO have a higher proportion of abdominal visceral fat and less thigh subcutaneous fat than individuals with MHO, which may contribute to elevated hs-CRP levels [26]. Genetic predisposition also plays a role in these phenotypic differences [47]. A knockout mouse model of the bromodomain-containing two genes showed that even on a regular diet, the mice became obese while remaining protected against insulin resistance, as indicated by normal glucose and low pro-inflammatory cytokine levels [48].

This study observed significant sex-related differences in the association between metabolic obesity phenotypes and hs-CRP levels. These results are in accordance with those of prior research showing that the association between obesity and inflammation is stronger in women than in men [17,34]. Additionally, previous studies have demonstrated that the association between MetS and elevated hs-CRP levels is more pronounced in women than in men [49,50,51]. Although the exact mechanism has been understudied, several mechanisms underlie these sex-related differences. First, women have a higher percentage of body fat than men with similar BMI levels [52], leading to elevated hs-CRP levels and increased CVD risk. Second, previous studies have demonstrated sex-based differences in leptin levels. Leptin, a hormone produced by adipocytes, is positively correlated with elevated CRP levels [53,54]. Higher leptin concentrations are associated with increased body fat and the female sex, which may partially account for the sex differences observed in the association between obesity and hs-CRP level [55].

This study has several limitations. First, our analysis was confined to hs-CRP levels, which limited our ability to assess the full spectrum of systemic inflammation. Therefore, future studies should encompass a broader array of inflammatory markers, such as IL-1β, IL-6, or TNF-α, to provide a more comprehensive understanding of the inflammatory processes underlying the different metabolic obesity phenotypes. Future studies should incorporate a broader panel of inflammatory markers to provide a more comprehensive assessment of systemic inflammation across the metabolic obesity phenotypes. Second, owing to the cross-sectional design, we were unable to draw a causal association between metabolic obesity phenotypes and hs-CRP levels. Longitudinal investigations are required to prospectively examine the trajectory of obesity phenotypes and their association with elevated hs-CRP levels. For instance, considering that the MHO type may transition to the MUO type over time, future studies should investigate whether such transitions are associated with an increase in levels of inflammatory markers. Third, our study was limited to the Korean population and may not be directly generalizable to other ethnicities or regions. For instance, it should be considered that the Asia-Pacific region utilizes a distinct BMI cutoff for obesity classification compared to that adopted by the World Health Organization. Fourth, an in-depth analysis considering participants’ age was not conducted, such as how the association between metabolic obesity phenotypes and hs-CRP levels varies across age groups, highlighting the need for further investigation.

Our study has some strengths. First, it leveraged a large-scale population-based sample of Korean adults, addressing the limitations of previous studies that were constrained by small sample sizes. This enables a more precise estimation of hs-CRP levels among the population across the different metabolic obesity phenotypes. Second, the measurements were conducted by healthcare professionals, thereby minimizing measurement errors. For instance, BMI was measured according to a standardized protocol rather than relying on self-reported data.

## 4. Materials and Methods

### 4.1. Study Sample

This cross-sectional study included participants from the 2015 to 2018 Korea National Health and Nutrition Examination Survey (KNHANES). The KNHANES is a population-based nationwide survey that includes a nationally representative sample of the general Korean population. To select this study participants, 192 administrative regions covering South Korea served as primary sampling units, and households in each region were systematically selected. Trained interviewers conducted household-visit health surveys and examinations for each household. Participants whose hs-CRP levels were measured during 2015–2018 KNHANES were included in this analysis. Individuals with hs-CRP levels exceeding 10 mg/L were excluded because these levels are indicative of acute inflammatory conditions, such as infections [56]. Consequently, a total of 21,112 adults aged 18 and older, with complete data, were included in this study. All of this study participants provided informed consent, which the Institutional Review Board of the Korea Disease Control and Prevention Agency authorized. As this study involved a secondary analysis of an anonymized, publicly available dataset, the Institutional Review Board of Severance Hospital approved this study with an exempt status (approval number: 4–2024–0953). The data are accessible at the homepage of the Korea Disease Control and Prevention Agency (https://knhanes.kdca.go.kr/knhanes/eng/index.do; accessed on 23 August 2024).

### 4.2. Metabolic Obesity Phenotype

#### 4.2.1. Obesity

The height of the participants was measured to the nearest 0.1 cm and weight to the nearest 0.1 kg while wearing light clothing. Height was measured using a Seca 274 (SECA, Hamburg, Germany) stadiometer, and weight was measured using a GL-6000-20 (G-Tech International Co., Uijeongbu-si, Gyeonggi-do, Republic of Korea) electronic balance following a standardized protocol. Body mass index (BMI) was calculated as weight divided by height squared (kg/m^2^). Based on the criteria used in the Asia-Pacific region, including Korea, obesity was defined as BMI ≥ 25 kg/m^2^ [57].

#### 4.2.2. Metabolic Phenotypes

The classification criteria for metabolic obesity phenotypes were based on the harmonized definition of MHO and MUO that is described in the previous literature [23,58]. According to the definition, MHO is defined as obesity in individuals who meet the criteria for obesity but do not meet any of the four components of MetS (excluding waist circumference). The four components of MetS, as defined by the National Cholesterol Education Program Adult Treatment Panel III (NCEP-ATP III), are as follows: (i) elevated triglycerides (≥150 mg/dL) or currently receiving medical treatment for elevated triglycerides; (ii) reduced high-density lipoprotein (HDL) cholesterol (<40 mg/dL for men and <50 mg/dL for women) or currently receiving medical treatment for reduced HDL cholesterol; (iii) high blood pressure (BP) (systolic BP ≥ 130 mm Hg and/or diastolic BP ≥ 85 mm Hg) or currently under treatment for elevated BP; and (iv) high fasting glucose (≥100 mg/dL) or currently under medical treatment for elevated glucose [59]. A harmonized definition of MHO is based on the notion that individuals who meet even one component of MetS cannot be considered “metabolically healthy.” MHO was defined as the presence of obesity (BMI ≥ 25 kg/m^2^) without meeting any of the four components of MetS. MUO was defined as obesity (BMI ≥ 25 kg/m^2^) in individuals who met at least one of the four MetS components. MUNO referred to individuals without obesity (BMI < 25 kg/m^2^) who met at least one of the four MetS components. MHNO refers to individuals without obesity (BMI < 25 kg/m^2^) and without meeting any of the four MetS components.

All blood samples were collected after 8 h of fasting. Samples were collected via venipuncture, immediately refrigerated, and transported to the central laboratory in cold storage for 24 h. Triglyceride, HDL cholesterol, and glucose levels were analyzed using the enzyme method with a Hitachi Automatic Analyzer 7600-210 (Hitachi, Tokyo, Japan). BP was measured after the participants had rested for at least 5 min using a mercury sphygmomanometer (Baumanometer Wall Unit 33, 0850; Baum Co., Inc., Copiague, NY, USA). Measurements were conducted three consecutive times, and the average of the second and third measurements was used for the analysis [60].

### 4.3. Measurement of hs-CRP

Blood samples were collected in 3-mL EDTA-coated tubes (BD Vacutainer, Franklin Lakes, NJ, USA), and serum samples intended for hs-CRP analysis were stored in refrigerated conditions at temperatures between 2 and 8 °C. All laboratory analyses were conducted within 24 h of sample collection. Serum hs-CRP levels were measured using immunoturbidimetry on a Cobas analyzer (**Roche** Diagnostics, Mannheim, Germany) with Roche Cardiac C-Reactive Protein High-Sensitive Reagent (Roche, Germany). The lower detection limit (LOD) was 0.15 mg/L, and half of the LOD value was used for values below the LOD.

### 4.4. Statistical Analysis

The distribution of hs-CRP values across metabolic obesity phenotypes was illustrated using a box plot, and comparisons between groups were performed using Wilcoxon’s Rank Sum Test. A Bonferroni correction was applied to account for multiple comparisons. Additionally, the correlation between BMI and hs-CRP levels was calculated using Pearson’s correlation coefficient. Subsequently, the association between the metabolic obesity phenotypes and hs-CRP levels was examined using linear regression models. To achieve a normal distribution of the dependent variables, hs-CRP values were log-transformed, which is in line with prior studies [61,62,63]. As a result, the association between metabolic obesity phenotypes and hs-CRP levels was expressed as a percentage change with 95% confidence intervals (CIs). Univariate and multivariate models were fitted sequentially. In the multivariate model, the following sociodemographic features of this study participants were adjusted for in the regression models: sex, age, educational level (categorized into middle school or below, high school, college or above), income level (categorized into lowest, low, high, and highest based on the quantile values of total household income for each year), marital status (categorized into married, unmarried, or others), employment status (categorized into employed or unemployed), smoking status (yes or no), and physical activity (yes or no). Regarding smoking status, participants were asked, “Do you currently smoke?” Those who responded “yes” were classified as smokers, and those who responded “no” were classified as non-smokers. Physical activity was assessed using the Korean version of the Global Physical Activity Questionnaire [64]. Following the World Health Organization guidelines, participants were classified as “yes” if they regularly engaged in at least 150 min of moderate-to-vigorous physical activity per week and “no” if they did not [65]. Next, we explored sex-based differences in the association between metabolic obesity phenotypes and log-transformed hs-CRP values by including interaction terms (metabolic obesity phenotype × sex). Subsequently, we conducted a sex-stratified analysis to examine how the association between metabolic obesity phenotypes and hs-CRP levels differed according to sex. Additionally, BMI was controlled for in all adjusted models.

For additional analyses, we applied alternative criteria for the metabolic obesity phenotype because prior studies have indicated that the association between metabolic obesity phenotypes and inflammatory markers may vary depending on the classification criteria. In this analysis, more lenient criteria for MHO were used, defining it as meeting 0 or 1 of the four MetS components [66,67]. Additionally, we classified hs-CRP level values as high CVD risk (≥3 mg/L) and low CVD risk following the classification criteria proposed by the American Heart Association [56]. Logistic regression models were employed to determine how metabolic obesity phenotypes were associated with high CVD risk. All statistical analyses and visualizations were performed using the R software for Windows (version 4.2.3; R Foundation for Statistical Computing, Vienna, Austria). Considering the systematic sampling procedure of the KNHANES, survey weights assigned to participants were reflected in the analyses using the “survey” package [68]. Statistical significance was defined as adjusted *p* < 0.05.

## 5. Conclusions

Our study showed that individuals with MUO and MHO exhibited elevated hs-CRP levels compared to those with MHNO. The MHO group had a lower hs-CRP level than the MUO group; however, the hs-CRP level was significantly higher than that of the MHNO group. Women exhibited a more pronounced increase in hs-CRP levels in both the MUO group.

## Figures and Tables

**Table 1:** Distribution of characteristics of the sample (*N* = 21,112).

	Overall	Metabolic Obesity Phenotypes			
		MHNO	MUNO	MHO	MUO
	*N* = 21,112	*N* = 5019	*N* = 8763	*N* = 856	*N* = 6474
Sex					
Male	9250 (43.8)	1574 (31.4)	3920 (44.7)	417 (48.7)	3339 (51.6)
Female	11,862 (56.2)	3445 (68.6)	4843 (55.3)	439 (51.3)	3135 (48.4)
Age					
19–25	1701 (8.1)	985 (19.6)	338 (3.9)	140 (16.4)	238 (3.7)
26–35	2690 (12.7)	1144 (22.8)	707 (8.1)	211 (24.6)	628 (9.7)
36–45	3898 (18.5)	1303 (26.0)	1300 (14.8)	200 (23.4)	1095 (16.9)
46–55	3933 (18.6)	809 (16.1)	1673 (19.1)	150 (17.5)	1301 (20.1)
56–65	4148 (19.6)	532 (10.6)	2080 (23.7)	93 (10.9)	1443 (22.3)
>65	4742 (22.5)	246 (4.9)	2665 (30.4)	62 (7.2)	1769 (27.3)
Education level					
Middle school or below	6413 (30.4)	517 (10.3)	3299 (37.6)	119 (13.9)	2478 (38.3)
High school	6929 (32.8)	1853 (36.9)	2770 (31.6)	327 (38.2)	1979 (30.6)
College or above	7770 (36.8)	2649 (52.8)	2694 (30.7)	410 (47.9)	2017 (31.2)
Income level					
Lowest	3809 (18.0)	407 (8.1)	1956 (22.3)	77 (9.0)	1369 (21.1)
Low	5116 (24.2)	1076 (21.4)	2161 (24.7)	206 (24.1)	1673 (25.8)
High	5900 (27.9)	1586 (31.6)	2275 (26.0)	303 (35.4)	1736 (26.8)
Highest	6287 (29.8)	1950 (38.9)	2371 (27.1)	270 (31.5)	1696 (26.2)
Marital status					
Married	17,526 (83.0)	3355 (66.8)	7837 (89.4)	600 (70.1)	5734 (88.6)
Unmarried or others	3586 (17.0)	1664 (33.2)	926 (10.6)	256 (29.9)	740 (11.4)
Employment type					
Employed	12,986 (61.5)	3248 (64.7)	5139 (58.6)	583 (68.1)	4016 (62.0)
Unemployed	8126 (38.5)	1771 (35.3)	3624 (41.4)	273 (31.9)	2458 (38.0)
Smoking					
Yes	3227 (15.3)	549 (10.9)	1408 (16.1)	126 (14.7)	1144 (17.7)
No	17,885 (84.7)	4470 (89.1)	7355 (83.9)	730 (85.3)	5330 (82.3)
Physical activity					
Yes	9382 (44.4)	2577 (51.3)	3596 (41.0)	485 (56.7)	2724 (42.1)
No	11,730 (55.6)	2442 (48.7)	5167 (59.0)	371 (43.3)	3750 (57.9)
BMI (kg/m^2^)					
Median (Q1, Q3)	23.7 (21.5, 26.0)	21.3 (19.8, 22.7)	22.7 (21.2, 23.9)	26.5 (25.6, 28.1)	27.1 (25.9, 28.9)

Values are presented as n (%) for categorical variables. MHNO, metabolically healthy non-obesity; MUNO, metabolically unhealthy non-obesity; MHO, metabolically healthy obesity; MUO, metabolically unhealthy obesity; BMI, body mass index.

**Table 2:** Log-linear regressions of the metabolic obesity phenotypes and hs-CRP levels among the overall sample.

	Univariate Model		Multivariate Model	
	% Change (95% CI)	*p*	% Change (95% CI)	*p*
Overall sample				
MHNO	Reference		Reference	
MUNO	52.0 (46.3–57.9)	<0.001	29.3 (24.1–34.6)	<0.001
MHO	94.0 (79.7–109.4)	<0.001	15.4 (6.4–25.2)	<0.001
MUO	130.3 (121.1–139.9)	<0.001	23.3 (16.4–30.5)	<0.001
Stratified by obesity				
MHNO	Reference		Reference	
MUNO	52.0 (46.3–57.9)	<0.001	25.7 (20.6–31.1)	<0.001
MHO	Reference		Reference	
MUO	18.7 (10.0–28.1)	<0.001	14.1 (5.5–23.4)	0.001
Stratified by metabolic dysfunction				
MHNO	Reference		Reference	
MHO	94.0 (79.7–109.4)	<0.001	19.3 (7.6–32.3)	0.001
MUNO	Reference		Reference	
MUO	51.6 (46.3–57.0)	<0.001	−5.2 (−9.9–−0.2)	0.042

MHNO, metabolically healthy non-obesity; MUNO, metabolically unhealthy non-obesity; MHO, metabolically healthy obesity; MUO, metabolically unhealthy obesity; hs-CRP, high-sensitivity C-reactive protein; CI, confidence interval.

**Table 3:** Sex-stratified analysis of the association between the metabolic obesity phenotypes and hs-CRP.

	Male (*N* = 9250)		Female (*N* = 11,862)	
	% Change (95% CI)	*p*	% Change (95% CI)	*p*
Overall sample				
MHNO	Reference		Reference	
MUNO	22.3 (14.7–30.3)	<0.001	30.2 (22.8–38.2)	<0.001
MHO	15.8 (2.6–30.7)	0.018	16.0 (6.5–26.4)	<0.001
MUO	12.5 (3.0–22.9)	0.009	22.8 (13.6–32.8)	<0.001
Stratified by obesity				
MHNO	Reference		Reference	
MUNO	17.8 (11.1–24.9)	<0.001	30.4 (23.9–37.3)	<0.001
MHO	Reference		Reference	
MUO	0.4 (−10.2–12.1)	0.949	38.7 (24.0–55.2)	<0.001
Stratified by metabolic dysfunction				
MHNO	Reference		Reference	
MHO	16.4 (−0.8–36.5)	0.062	21.3 (5.8–39.2)	<0.001
MUNO	Reference		Reference	
MUO	−8.1 (−14.4–−1.4)	0.019	0.8 (−6.8–9.0)	0.851

MHNO, metabolically healthy non-obesity; MUNO, metabolically unhealthy non-obesity; MHO, metabolically healthy obesity; MUO, metabolically unhealthy obesity; hs-CRP, high-sensitivity C-reactive protein; CI, confidence interval.

## Data Availability

The data are accessible at https://knhanes.kdca.go.kr/knhanes/eng/index.do (accessed on 23 August 2024).

## Supplementary Materials

The following supporting information can be downloaded at: https://www.mdpi.com/article/10.3390/ijms252111540/s1.
