# Supplementary material for: Systemic Inflammation Across Metabolic Obesity Phenotypes: A Cross-Sectional Study of Korean Adults Using High-Sensitivity C-Reactive Protein as a Biomarker
Source: Int J Mol Sci. 2024 Oct 27;25(21):11540. doi: 10.3390/ijms252111540 (PMC11547168; doi:10.3390/ijms252111540)
Supplement: Supplementary file 1 [file ijms-25-11540-s001.zip › ijms-3257065-supplementary.pdf]

Table S1 Models with interaction terms between the metabolic obesity phenotypes and sex

	**% change**	**95% CI**	***P***
MHNO			
MUNO	27.4	19.8–35.6	< 0.001
MHO	14.4	2.2–28.1	0.019
MUO	15.2	7.3–23.6	< 0.001
Female × MUNO	2.5	-4.7–10.1	0.506
Female × MHO	1.3	-13.2–18.1	0.873
Female × MUO	18.3	9.6–27.7	< 0.001

MHNO, metabolically healthy non-obesity; MUNO, metabolically unhealthy non-obesity; MHO, metabolically healthy obesity; MUO, metabolically unhealthy obesity; hs-CRP, high-sensitivity C-reactive protein; CI, confidence interval

Table S2. Multivariate logistic regression models on the association between the metabolic obesity phenotypes and elevated hs-CRP level (≥ 3mg/L)

	OR (95% CI)	*P*
**Overall**		
MHNO	Reference	
MUNO	2.03 (1.59–2.59)	< 0.001
MHO	1.71 (1.15–2.54)	0.008
MUO	1.61 (1.17–2.21)	0.003
**Male**		
MHNO		
MUNO	1.59 (1.11–2.27)	0.012
MHO	1.63 (0.92–2.91)	0.096
MUO	1.00 (0.63–1.58)	0.993
**Female**		
MHNO		
MUNO	2.58 (1.86–3.59)	< 0.001
MHO	1.74 (1.00–3.03)	0.049
MUO	2.96 (1.94–4.52)	< 0.001

MHNO, metabolically healthy non-obesity; MUNO, metabolically unhealthy non-obesity; MHO, metabolically healthy obesity; MUO, metabolically unhealthy obesity; hs-CRP, high-sensitivity C-reactive protein; OR, odds ratio; CI, confidence interval

Table S3. Multivariate linear regression models of the association between the metabolic obesity phenotypes and hs-CRP levels based on alternative classification criteria

	**% change**	**95% CI**	***P***
**Overall**			
MHNO	Reference		
MUNO	24.0	18.9–29.4	< 0.001
MHO	7.8	1.7–14.1	0.011
MUO	12.4	6.6–18.5	< 0.001
**Male**			
MHNO	Reference		
MUNO	20.0	13.4–26.9	< 0.001
MHO	2.7	-5.5–11.7	0.525
MUO	7.5	-0.5–16.1	0.066
**Female**			
MHNO	Reference		
MUNO	30.2	22.8–38.2	< 0.001
MHO	16.0	6.5–26.4	0.001
MUO	22.8	13.6–32.8	< 0.001

MHNO, metabolically healthy non-obesity; MUNO, metabolically unhealthy non-obesity; MHO, metabolically healthy obesity; MUO, metabolically unhealthy obesity; hs-CRP, high-sensitivity C-reactive protein; CI, confidence interval

Table S4 Log-linear regressions of the metabolic obesity phenotypes and hs-CRP levels among the overall sample without adjustment for BMI

	Multivariate model	
	**% change (95% CI)**	***P***
**Overall sample**		
MHNO	Reference	
MUNO	42.7 (37.0–48.6)	< 0.001
MHO	89.7 (75.7–104.9)	< 0.001
MUO	114.9 (105.9–124.4)	< 0.001
**Stratified by obesity**		
MHNO	Reference	
MUNO	33.2 (27.7–38.9)	< 0.001
MHO	Reference	
MUO	26.8 (17.0–37.5)	< 0.001
**Stratified by metabolic dysfunction**		
MHNO	Reference	
MHO	85.0 (71.3–99.7)	< 0.001
MUNO	Reference	
MUO	51.2 (46.0–56.6)	< 0.001

Table S5. Sex-stratified analysis of the association between the metabolic obesity phenotypes and hs-CRP without adjustment for BMI

	Male (N = 9,250)		Female (N = 11,862)	
	**% change (95% CI)**	***P***	**% change (95% CI)**	***P***
**Overall sample**				
MHNO	Reference		Reference	
MUNO	35.6 (27.4–44.4)	< 0.001	48.7 (41.3–56.4)	< 0.001
MHO	80.7 (61.9–101.7)	< 0.001	97.1 (77.0–119.4)	< 0.001
MUO	88.4 (77.3–100.2)	< 0.001	155.1 (140.0–171.2)	< 0.001
**Stratified by obesity**				
MHNO	Reference		Reference	
MUNO	27.8 (19.8–36.3)	< 0.001	37.8 (30.8–45.3)	< 0.001
MHO	Reference		Reference	
MUO	12.2 (-0.1–25.9)	0.051	53.4 (37.0–71.8)	< 0.001
**Stratified by metabolic dysfunction**				
MHNO	Reference		Reference	
MHO	80.3 (61.4–101.5)	< 0.001	92.5 (72.6–114.6)	< 0.001
MUNO	Reference		Reference	
MUO	38.7 (32.3–45.4)	< 0.001	72.6 (63.9–81.7)	< 0.001
